# Immune-privileged cord blood-derived endothelial colony-forming cells: advancing immunomodulation and vascular regeneration

**DOI:** 10.1007/s10456-025-09973-9

**Published:** 2025-03-06

**Authors:** David M. Smadja, Yanis Berkane, Nun K. Bentounes, Jeanne Rancic, Audrey Cras, Cécile Pinault, Marie Ouarne, Elise Paucod, Walid Rachidi, Alexandre G. Lellouch, Maxime Jeljeli

**Affiliations:** 1 Université Paris Cité, INSERM U970, Paris Cardiovascular Research Center, Paris, France; 2 Hematology Department, AP-HP, Georges Pompidou European Hospital, Paris, F-75015 France; 3 Department of Plastic, Reconstructive and Aesthetic Surgery, Hôpital Sud, CHU Rennes, University of Rennes, Rennes, France; 4 SITI Laboratory, UMR INSERM 1236, Rennes University Hospital, Rennes, France; 5 Center for Engineering in Medicine and Surgery, Massachusetts General Hospital, Harvard Medical School, Boston, MA USA; 6 Cell Therapy Department, AP-HP, Saint-Louis Hospital, Paris, F-75010 France; 7 Univ. Grenoble Alpes, CEA, INSERM, IRIG-BGE UA13, Grenoble, 38000 France; 8 Department of Plastic, Reconstructive and Aesthetic Surgery, Cedars Sinai Hospital, Los Angeles, USA

**Keywords:** ECFC, Regeneration, Endothelial cells, Immunogenicity, Immunomodulation, Artificial skin, Organoid

## Abstract

Cord blood-derived endothelial colony-forming cells (CB-ECFCs) hold significant promise for regenerative medicine due to their unique vasculogenic and immunomodulatory properties. These cells exhibit a superior proliferative capacity, robust ability to form vascular networks, and lower immunogenicity compared to adult and embryonic stem cell-derived counterparts. The immune-privileged characteristics of CB-ECFCs, including reduced expression of pro-inflammatory mediators and tolerance-inducing molecules such as HLA-G, further enhance their therapeutic potential. Their low immunogenicity minimizes the risk of immune rejection, making them suitable for allogenic cell therapies. Their application extends to complex tissue engineering and organ revascularization, where their ability to integrate into three-dimensional scaffolds and support vascular tree formation represents a significant advancement. Moreover, CB-ECFCs’ capability to adapt to inflammatory stimuli and retain immunological memory highlights their functional versatility in dynamic microenvironments. This review highlights the remarkable ontogeny of ECFCs while unveiling the unparalleled potential of CB-ECFCs in revolutionizing regenerative medicine. From pre-vascularizing engineered tissues and organoids to pioneering cell-based therapies for cardiovascular, dermatological, and degenerative diseases, CB-ECFCs stand at the forefront of cutting-edge biomedical advancements, offering unprecedented opportunities for therapeutic innovation. By leveraging their vasculogenic, immune-regulatory, and regenerative capacities, CB-ECFCs offer a robust alternative for addressing the challenges of vascular repair and organ engineering. Future research should focus on unraveling their transcriptomic and functional profiles to optimize clinical applications and advance the field of regenerative medicine.

## Introduction

The field of regenerative medicine has made significant strides in recent years, with the potential of allogenic cell therapy emerging as a promising avenue for treating a variety of conditions [1]. Among the various cell types under investigation, endothelial colony-forming cells (ECFCs) derived from cord blood have garnered particular interest due to their unique vasculogenic properties and potential therapeutic applications [2]. These cells are notable for their ability to form blood vessels and their role in vascular repair, which positions them as a critical component in developing effective cell-based therapies. After years of inconsistency, consensus has been recently obtained about their isolation and characterization from cord and adult blood [2–4].

Cord blood, a rich source of hematopoietic stem cells, also provides a reservoir of ECFCs with distinct advantages over other sources [5]. These cells are less mature, exhibit higher proliferative capacity and clonogenic properties, and demonstrate a robust ability to form vascular networks. Moreover, the use of cord blood-derived ECFCs (CB-ECFCs) introduces fewer ethical concerns compared to embryonic stem cells (ESCs). It also presents a lower risk of graft-versus-host disease compared to adult stem cells, highlighting their potential for safer and more effective clinical applications. A pivotal aspect of using ECFCs in allogenic cell therapy could be their immune privilege. The concept of immune privilege refers to the ability of certain cells or tissues to evade detection and destruction by the host immune system. This property is particularly beneficial for allogenic transplants, as it can reduce the likelihood of immune rejection and improve the overall success rates of cell-based therapies. Understanding and harnessing the mechanisms of immune privilege in CB-ECFCs could pave the way for more widespread and successful implementation of these cells in clinical settings.

This paper aims to explore the characteristics and therapeutic potential of CB-ECFCs, with a focus on ontogeny, immune-privileged status and bioengineering applications. We will review current research on the mechanisms that confer an immune privilege to these cells, discuss their advantages over other cell types, and evaluate the feasibility of their use in allogenic cell therapy. By delving into these aspects, we hope to provide a comprehensive overview of this field and outline the potential pathways toward translating this promising research into clinical practice.

## ECFCs: a human vasculogenic cell type usable for vessel reconstruction

### ECFC ontogeny

The identification of adult endothelial progenitor cells (EPCs) in humans in 1997 dramatically changed the understanding of postnatal blood vessel formation [6]. Within the different EPC subtypes, ECFCs also named as non-hematopoietic EPCs [7] have gained recognition for their notable ability to promote vasculogenesis and their culture and isolation have been standardized recently [3]. Despite the substantial interest in their application as a cell therapy product or as a component of liquid biopsies, the exact tissue and molecular origins of ECFCs remain contentious. While Lin et al., using a sex-mismatch human bone marrow transplantation model, reported that ECFCs do originate from bone marrow [8], Fujisawa et al. demonstrated the opposite more recently in five male recipients who had received allogenic bone marrow transplants from female donors to treat hematological disorders [9].

ECFCs were initially identified in peripheral blood and cord blood-derived mononuclear cells cultures [5, 8, 10]. Subsequent studies demonstrated that ECFCs can arise from CD34 + cells, as well as from specific subpopulations expressing CD146+/CD34+/CD45+/CD133 + or CD177 + markers [11–13]. Subsequent research conducted by Case et al. and Timmermans et al. demonstrated that although CD34^+^/CD45^−^ cells possess the ability to differentiate into ECFCs, they lack the capacity to generate hematopoietic colonies [14, 15]. This was later confirmed by Tura et al., who identified CD34+/CD146+/CD133 − cells as the precursor population for ECFCs [16]. However, the exact stem cell at the origin of ECFCs is quite elusive. thus, several descriptions of ECFCs Originating from CD34 + are convincing at this time. Lin et al. [17] provides crucial insights into the developmental origins of ECFCs. Their study demonstrates that ECFCs originate specifically from CD34 + cells in umbilical cord blood and adult peripheral blood. CD34bright mononuclear cells in cord blood are the primary source of ECFCs, confirming their distinct endothelial lineage. Lineage tracing experiments in mice reveal that ECFCs are not derived from the bone marrow hematopoietic system but rather from vascular endothelium. Unlike hematopoietic EPCs, which play only proangiogenic roles, ECFCs possess real vasculogenic capacity, forming functional blood vessels in vivo [10]. The study demonstrates that ECFCs are a distinct subset of postnatal, tissue-derived vascular progenitors. Another subset of CD34 + cells has been described to give rise to ECFCs like cells. Indeed, very small embryonic-like stem cells (VSELs), identified in both humans and mice, have been proposed to give rise to differentiate into multiple lineages [18] but in particular in endothelial cells in vivo [19], in vitro [20] but also in ECFCs-like cells after differentiation in vitro [21]. VSELs have been first described as CD133^+^ small dormant stem cells and are linked to migratory primordial germ cells. They have been found in bone marrow and other organs, such as the heart and lungs [18]. The pluripotent capacity of VSELs presents a promising avenue for resolving the debate about bone marrow stem cell reservoirs and the conflicting data surrounding endothelial ontogeny and the Hemangioblasts hypothesis in humans. By bridging the gap between the developmental plasticity of stem cells and the observed functional properties of ECFCs, VSELs could unify disparate findings, providing a comprehensive framework to understand the ontogeny and potential reservoirs of endothelial progenitor cells. This resolution could advance both theoretical knowledge and practical applications in regenerative medicine.

Despite their potential in clinic, traditional methods of isolating and expanding CD34 + cells have been limited by scalability and reproducibility. To overcome this, Pr Hénon’s team developed an automated GMP-compliant expansion platform designed for large-scale production of expanded CD34 + cells [22] named ProtheraCytes, ensuring a controlled and standardized therapeutic product. The ProtheraCytes manufacturing process involves several key steps. CD34 + cell mobilization and collection is performed using granulocyte-colony stimulating factor to mobilize CD34 + cells from the bone marrow into the peripheral blood, followed by collection through leukapheresis. The cells then undergo a nine-day expansion process within a closed, aseptic bioreactor system known as StemXpand, increasing CD34 + cell numbers by approximately sixteen- to nineteen-fold. The final product is subjected to immunoselection and quality control, ensuring high viability, purity, and paracrine secretion potential while complying with Good Manufacturing Practice standards [22]. ProtheraCytes exhibit a gene expression profile indicative of early endothelial differentiation potential [23] 10.1038/s41598-023-47079-8. Transcriptomic analysis reveals upregulation of genes associated with mesoderm and endothelial commitment [23]. These expanded cells also contain an enriched content of CD34 + VSELs, which retain their pluripotency and probably contribute to endothelial differentiation. Thus, protheraCytes represent a new class of expanded CD34 + progenitor cells, designed to harness the regenerative potential of endothelial lineage cells and confirm that CD34 + cells have potential for endothelial lineage 10.1038/s41598-023-47079-8 [24].

Moreover, tissue-Resident Endothelial Stem Cells expressing CD157 (endothelial protein C receptor; EPCR) have been described to be a good candidate for ECFCs. Indeed, two significant papers by Yoder’s group [25, 26] provide compelling evidence that ECFCs originate from this resident endothelial populations. Wakabayashi et al. describe a population of CD157 + vascular endothelial stem cells (VESCs) found in large arteries and veins across multiple organs. Using genetic lineage tracing in mice, they demonstrated that these cells exhibit clonal proliferative capacity and are able to generate new ECs. They maintain large blood vessels and sinusoids for over a year, indicating a role in long-term vascular homeostasis. They expand and regenerate entire vasculature structures in response to injury. Furthermore, these cells can be transplanted to rescue bleeding phenotypes, confirming their regenerative capabilities. The discovery of CD157 as a marker for VESCs is particularly important, as it provides a means to prospectively isolate and study these cells, offering strong in vivo evidence that the vascular system contains its own stem cell reservoir, rather than depending solely on circulating progenitors or embryonic-like progenitors. Building on this work, Naito et al. [26] established a robust protocol for isolating tissue-resident VESCs from the mouse liver. Their findings confirm that CD157 + VESCs exist within liver blood vessels and can be efficiently isolated using fluorescence-activated cell sorting (FACS). These cells exhibit self-renewal properties and contribute to new blood vessel formation in an experimental setting. Their angiogenic potential is greater than that of mature endothelial cells, suggesting that vascular homeostasis relies on a subpopulation of stem-like cells within vessels. These two studies strongly indicate that ECFCs are likely derived from resident vascular progenitors rather than a single embryonic progenitor pool. Adult vasculature harbors its own regenerative endothelial cell population, reducing reliance on circulating or bone marrow-derived progenitors. CD157 + VESCs may serve as a therapeutic target for vascular repair. Bhagwani et al. [27] demonstrate that primitive endothelial cells can undergo clonal selection and contribute to vascular disease, particularly in pulmonary hypertension (PH). Their study found that CD117 + primitive endothelial cells proliferate abnormally, leading to occlusive vascular pathology. These cells retain endothelial characteristics but also show mesenchymal differentiation potential. This suggests that certain endothelial clones can drive pathological vascular remodeling. Lin et al. [28] identified ABCG2-expressing clonal endothelial cells as a unique population that maintains vascular homeostasis and contributes to endothelial turnover. These cells exhibit higher proliferative potential than mature endothelial cells. They are distinct from embryonic-like progenitors and instead represent a local vascular stem cell pool. Their findings reinforce the notion that vascular progenitors reside within adult blood vessels, making the small embryonic stem cell hypothesis less tenable.

Mesenchymal stem cells (MSCs) derived from adult bone marrow have been widely studied for their regenerative potential due to their ability to differentiate into various mesodermal lineages, including osteogenic, chondrogenic, adipogenic and perivascular lineages. However, despite extensive research, there is no strong evidence to support the notion that adult bone marrow-derived MSCs can differentiate into fully functional endothelial cells capable of participating in vasculogenesis or angiogenesis in vivo [29, 30]. In contrast, MSCs with a more immature phenotype, such as those derived from perinatal tissues, seem to exhibit a greater degree of plasticity and appear to have a broader differentiation potential. Wharton’s jelly-derived MSCs, which are obtained from the umbilical cord, have been shown to express both mesenchymal and endothelial markers more readily compared to their adult bone marrow counterparts. These cells can be induced to differentiate into endothelial-like cells [31, 32]. Their perinatal origin suggests that they retain an epigenetic profile that is more permissive to endothelial differentiation compared to adult MSCs, which may have undergone further lineage restriction. Another subset of MSCs with endothelial differentiation potential includes those isolated from CD133 + progenitor populations [19]. Furthermore, MSC-like cells derived from vascular tumors such as infantile hemangiomas have demonstrated the ability to undergo endothelial differentiation [33, 34]. These findings suggest that MSCs originating from sources that retain a closer developmental proximity to vascular progenitors may be more amenable to endothelial differentiation compared to bone marrow-derived MSCs. Gil et al. [35] demonstrate that mesodermal cell from induced pluripotent stem cells (iPSCs) can differentiate into ECFC-like cells, restoring vascular function in diabetic mice. Moreover, Pal et al. [36] introduce an entirely different model, proposing that fibroblasts can transdifferentiate into endothelial-like cells following injury. Using single-cell RNA sequencing, they found that fibroblasts reprogram into endothelial-like cells after miR-200b inhibition. This vasculogenic fibroblast subset contributes to wound healing and neovascularization. This challenges the assumption that ECFCs must derive from endothelial precursors, as non-endothelial cells may contribute to endothelial lineage plasticity. Their findings indicate that ECFCs may emerge from non-traditional sources, further complicating the assumption that they originate solely from small embryonic-like stem cells.

### ECFC characterization

The characterization of ECFCs requires precise criteria to ensure reproducibility and consistency as previously described [3, 4]. These colonies should present a well-defined structure with a clonogenic nature, displaying a cobblestone morphology and comprising more than fifty adherent cells. Their abundance is quantified as the number of colonies per ten million mononuclear cells seeded, providing a standardized metric for comparison. Isolation efficiency is a crucial parameter, with expert laboratories achieving a success rate of seventy to seventy-five% in deriving these cells from the peripheral blood of healthy individuals. However, variability in donor samples must be acknowledged, as certain cases result in the absence of ECFC colonies. This outcome, referred to as “zero colonies,” is an essential data point that must be documented. Beyond initial isolation, the expansion potential of these cells should be assessed through both passage number and population doubling time, offering deeper insights into their proliferative capacity. Additionally, studies should systematically report the duration of culture, starting from the day of the original mononuclear cell isolation, known as day zero. By adhering to these rigorous characterization standards, research on ECFCs can maintain a high level of reliability, paving the way for advancements in vascular biology and regenerative medicine.

**Fig. 1:**
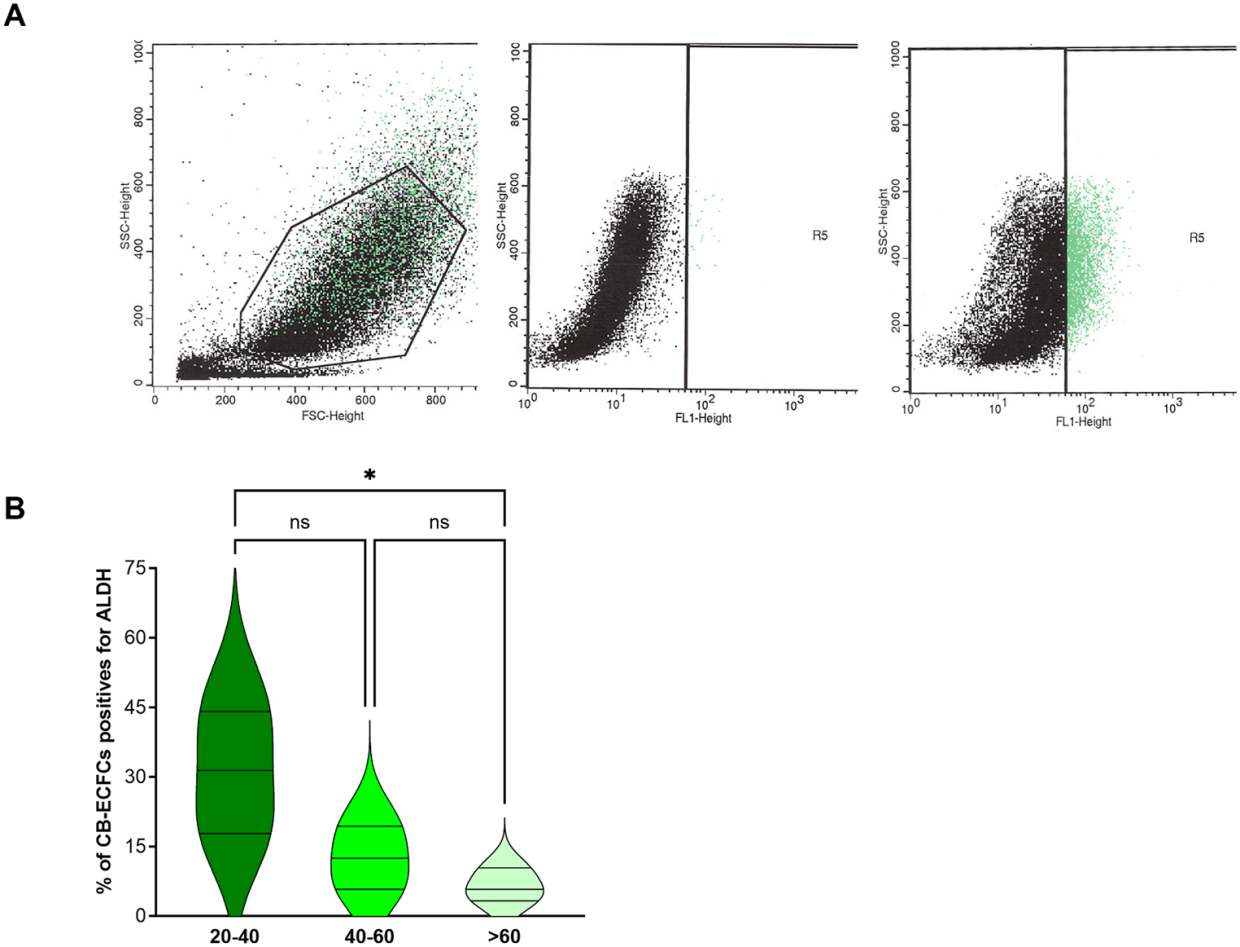
Aldehyde dehydrogenase (ALDH) activity in CB-ECFCs during in vitro expansion. Quantification of ALDH activity. ALDH activity was measured using the Aldefluor reagent (StemCell Technologies) following the manufacturer’s instructions. **A** concentration of 0.625 µg/mL Aldefluor substrate was added to 1 million CB-ECFCs in Aldefluor assay buffer and incubated for 30 min at 37 °C, converting the substrate to a fluorescent product. To ensure specificity, a portion of the Aldefluor-stained cells was treated with 5 µL of 1.5 mM diethylaminobenzaldehyde (DEAB), a specific ALDH inhibitor, as a control (center panel). After Aldefluor treatment, the cells were stained with fluorochrome-conjugated antibodies and the fluorescence was quantified using an Accuri C6 PLUS (BD Biosciences) (right panel). **B**:Evolution of ALDH activity of CB-ECFCs. CB-ECFCs were cultured in endothelial growth medium 2 (EGM2, Lonza Paris, France) and the quantification of ALDH activity was performed from 20 days after starting of the culture for several weeks. A significant decrease of ALDH-positive cells proportion was observed along culture and passages in culture. The x-axis represents the number of days after MNC culture

ECFCs are characterized by the presence of specific endothelial markers, including CD31, CD34 [37, 38], intracellular CD133 [39], VE-Cadherin, vascular endothelial growth factor receptor 2 (KDR), von Willebrand factor (VWF) in Weibel Palade bodies and MCAM (CD146) [40]. They also express Aldehyde Dehydrogenase (ALDH) [41–43]. ALDH is a group of enzymes that plays a crucial role in detoxifying aldehydes thus inducing protection against oxidative stress. ALDH is also involved in retinoic acid biosynthesis from retinaldehyde, which is crucial for cellular differentiation, and immune function and has an important role in maintaining “stemness,” the stem cells ability to self-renew and differentiate [44]. As shown in Fig. 1, ALDH is expressed by CB-ECFCs, and its expression decreases along in vitro expansion. This novel observation of ALDH expression and its decline with in vitro expansion adds a new dimension to understanding the biology of ECFCs. It suggests a potential link between ALDH activity and the maintenance of stemness or functional capabilities in ECFCs. This finding could provide valuable insights into optimizing culture conditions for therapeutic applications, preserving the regenerative potential of ECFCs, and further exploring the role of ALDH as a marker for stemness and cellular functionality. Finally, an overexpression of neuregulin-1 (NRG1) in ECFCs compared to mature endothelial cells has also been described [45]. Unlike classic endothelial cells, ECFCs actively secrete bioactive NRG1, promoting cardiomyocyte survival, proliferation, and engraftment through the PI3K/Akt signaling pathway. This effect is absent in mature endothelial cells. In co-culture experiments and in vivo models, ECFCs enhanced cardiomyocyte resistance to apoptosis and improved cardiac cell retention, whereas mature endothelial cells failed to provide similar benefits. Silencing NRG1 in ECFCs abolished these protective effects, confirming its essential role. This study establishes ECFCs as distinct from mature endothelial cells due to their regenerative potential, making them promising candidates for cardiovascular therapy, particularly in cardiac repair and ischemic heart disease treatment.

### ECFC properties

In addition to the difference between the number of colonies obtained [46], CB-ECFCs exhibit superior proliferative capacity compared to their adult blood-derived counterparts [5, 47, 48] and demonstrate a remarkable resistance to apoptosis and stemness marker expression, distinguishing them from HUVECs [12, 39, 49], probably linked to, at least in part, longer telomeres [50]. CB-ECFCs have been observed to promote vessel formation [51], even better than human Embryonic Stem Cell-Endothelial Cells (hESC-EC) [52].

One of their key mechanisms of action is their direct integration into host tissues, where they actively participate in vessel formation, a capability that sets them apart from hematopoietic EPCs [5] and bone marrow-derived mesenchymal stem cells [30]. Unlike these other cell types, ECFCs can engraft directly into tissues and contribute to the formation of human vasculature in murine models of hind limb ischemia, underscoring their therapeutic potential in vascular regeneration [5, 10, 40]. Beyond direct incorporation, another crucial mechanism underlying ECFC functionality is their paracrine influence. Initially, ECFCs were believed to lack significant secretory activity, particularly in comparison to hematopoietic EPCs, as they produced neither vascular endothelial growth factor-A nor substantial levels of interleukin-8 [10]. However, further research has revealed that under inflammatory, stress, or senescent conditions, ECFCs possess a dynamic secretion profile, releasing interleukin-8 and other chemokines [53, 54], such as CXCL and CCL molecules [55], to actively recruit immune cells. Additionally, they facilitate perivascular cell recruitment through the secretion of platelet-derived growth factor-BB [56], thereby enhancing cellular engraftment and tissue integration. Their secretory capacity extends to the release of microvesicles and exosomes, which are believed to contribute significantly to their regenerative effects [57–59]. Another pivotal mechanism involves the direct supportive role ECFCs play in mesenchymal stem cell function. These interactions, observed across various mesenchymal stem and progenitor cell origins, suggest that ECFCs promote the differentiation of mesenchymal precursors into bona fide perivascular cells through a Notch/Jagged-1 signaling pathway [60]. This crosstalk is crucial for optimizing vascular stabilization and regeneration in ischemic conditions. Lastly, ECFCs exhibit a unique adhesive capacity that facilitates perivascular cell attachment via an endoglin-dependent mechanism [61]. When mesenchymal stem cells are co-administered in models of hind limb ischemia, endoglin binding by ECFCs accelerates perivascular cell engraftment, leading to enhanced vascular repair and expedited recovery [61]. This adhesive interaction represents a critical feature of ECFC-mediated vascular regeneration, further solidifying their role as a cornerstone in vascular therapeutic strategies. Nonetheless, numerous preclinical studies on vasculogenesis using human ECFCs have shown that for successful engraftment, it is often necessary to co-transplant perivascular support cells, such as mesenchymal stromal cells (MSCs) [29, 61–63]. Given the challenges of using multiple cell types in clinical settings, such as the complexity and regulatory hurdles associated with ECFCs and MSCs, alternative approaches are necessary. Lin and colleagues offer a promising solution by demonstrating the pivotal role of mitochondrial transfer from MSCs to endothelial cells (ECs) via tunneling nanotubes (TNTs). This mitochondrial transfer is crucial for the successful integration of ECFCs, suggesting that replacing MSCs with isolated mitochondria might simplify the clinical application while retaining the therapeutic benefits [64, 65].

## Beyond vascular regeneration, the immunomodulatory properties of ECFCs

The use of induced Pluripotent Stem-derived endothelial cells (iPS-ECs) in regenerative medicine offers significant advantages, including the ability to engineer them to match the genetic profile of the patient and thus minimize the risk of immune rejection [2]. However, ECFCs, particularly those derived from CB exhibit a low immunogenicity level, in addition to their increased vasculogenic potential, making them highly valuable in this context. Indeed, the first description of EPC-derived ECs in 2010 demonstrated low levels of major histocompatibility complex (MHC) class I (MHC I) and no constitutive expression of MHC class II (MHC II) in contrast to mature ECs in rats [66]. MHC I and MHC II are responsible for distinct immune responses. MHC I expression on allogeneic tissue is responsible for the activation of cytotoxic CD8^+^ T cells [67] and complement-mediated lysis upon alloantibodies binding [68] while antigen recognition via MHC II is critical for the activation of allogeneic CD4^+^ T cell and the initiation of the direct pathway of allorecognition [69].

Interestingly, endothelial cells derived from endothelial progenitor cells (EPC-derived ECs) in rats exhibit low expression of MHC class I molecules. This low expression is linked to a reduced susceptibility to attack by pre-activated allospecific cytotoxic CD8 + T lymphocytes and complement-mediated lysis [66]. Moreover, upon stimulation with interferon-gamma (IFN-γ), EPC-derived ECs upregulated MHC I but marginally upregulated MHC II compared to mature ECs [66]. Therefore, ECFCs low expression of MHC molecules makes them less likely to be recognized and attacked by the immune system and limits their ability to activate the adaptive immune system through the antigen presentation pathway. Indeed, despite their allogeneic origin, when EPC-derived ECs were seeded into acellular aortic grafts and transplanted into rats, the grafts exhibited excellent endothelialization and induced mild inflammatory responses without signs of rejection [66]. This suggests that EPC-derived ECs can be used effectively in vascular reconstruction without eliciting a strong immune response.

The immune function of mature endothelial cells has been clearly established, notably their capacity to recognize and respond to stimuli such as proinflammatory cytokines IL-6, IL-1β and tumor necrosis factor alpha (TNF-α), but also pathogen-associated molecular patterns, through the expression of different Toll-like receptors (TLRs) [70]. ECFCs have been shown to inherently express the full spectrum of TLRs, ranging from TLR1 to TLR10, but data on their functional role in ECFC are still underexplored [71]. TLR3 recognizes double-stranded RNA (dsRNA), a molecule associated with viral infections and cellular stress, and its stimulation in CB-ECFCs induces the release of pro-inflammatory cytokines including TNF-α, IL-6, and IL-8, and the expression of ICAM-1 and VCAM-1 adhesion molecules [72]. Additionally, TLR3 stimulation in CB-ECFCs impairs their proliferation and angiogenic capacity, delaying ischemia recovery in a hind-limb ischemia mouse model [72]. This effect has been corroborated in an apolipoprotein E-deficient model of atherogenesis where TLR3 stimulation in cultured endothelial progenitor cells reduced their migration capacity while increasing the apoptosis rate and their production of reactive oxygen species. Importantly, the adoptive transfer of TLR3-stimulated ECFCs hampered reendothelialization in a model of artery injury, further confirming the role of TLR3 in endothelial dysfunction [73].

In contrast, stimulation with lipopolysaccharide (LPS) upregulated TLR2 mRNA expression and increased IL-6 production in isolated CB-ECFCs, but it did not impact their growth or differentiation. Interestingly, TLR2 has been implicated in mobilizing endothelial progenitors from the bone marrow into circulation in a murine model of periodontal infection [74]. Furthermore, TLR2 signaling appears to play a critical role in tissue regeneration by modulating angiogenesis and oxidative stress during inflammatory conditions [75]. These findings highlight the distinct roles of TLR3 and TLR2 in endothelial progenitor cell biology and vascular function. TLR3 exerts anti-angiogenic effects and may impede regenerative processes, whereas TLR2 appears to facilitate regenerative outcomes through the modulation of inflammation and angiogenesis. Collectively, these data indicate that TLRs, particularly TLRs 2, 3 and 4 are functionally active in ECFCs and can influence angiogenic processes and the response to inflammatory stimuli. Further studies are needed to decipher the specific roles of TLRs subsets in ECFCs, particularly how they modulate angiogenic function and respond to various microenvironmental factors, including inflammation, infections and allogeneic conditions. Cell surface markers, such as CD34, play a key role in cell recognition, immune regulation, and intercellular communication [76].

CD34, a well-known surface marker associated with hematopoietic and endothelial progenitor cells, has been increasingly recognized for its functional relevance in modulating endothelial and immune cell behavior [7]. A recent study published in 2023 investigated the role of CD34 expression on ECFCs and endothelial cells and its impact on immune cell responses [77]. Using transcriptomic and functional analysis, the authors showed that CD34^− high^ ECFCs exhibited a lower proliferation rate, but higher secretion levels of IL-33 and Angiopoietin 2 compared to CD34^low^ ECFCs. Angiopoietin is a proangiogenic cytokine that drives vascular remodeling through effects on Tie2 receptor signaling in endothelial cells [78]. Interestingly, it has been shown to promote regulatory T cells (Treg) expansion through the stimulation of TIE2 expressing monocytes [79]. The co-culture of CD34 ^high^ ECFCs with naïve peripheral blood mononuclear cells (PBMC) strongly induced the differentiation of Tregs through the IL-33/ST2 axis [77]. IL-33 is produced in response to tissue injury and inflammation [80] and has been recently shown to induce Tregs expansion that controls the inflammatory response without altering the adaptive immune cells’ function [81]. In conclusion, the ability of ECFCs CD34^+^ to produce IL-33 and Angiopoietin 2 highlights their potential role in promoting immune tolerance in response to tissue injury and inflammation.

The activation of the TNF-α/TNFR2 signaling pathway has also been recently described as an additional mechanism involved in the immunoregulatory function of ECFCs [82]. TNF receptor type II (TNFR2) is preferentially expressed by immunosuppressive cells, including Tregs, and myeloid-derived suppressor cells (MDSCs).Its activation plays an important role in attenuating pro-inflammatory responses and promoting tissue regeneration [83]. Importantly, priming ECFCs with an appropriate dose of TNF-α could effectively upregulate TNFR2 expression and their immunoregulatory function without an excessive increase of the pro-inflammatory TNFR1 subset, indicating that the ECFCs respond to the inflammatory milieu to subsequently control inflammation-related tissue injury [84]. Of note, the immunosuppressive effect of ECFCs is independent of Tregs, as they did not increase Foxp3 expression in conventional T cells [82]. This observation is aligned with the lack of IL-10 and TGFβ production by T cells following co-culture with either CB-ECFCs or AB-ECFCs [82].

In addition to promoting immunoregulatory pathways, further studies have shown that human CB-ECFCs significantly express less pro-inflammatory mediators, such as arachidonate 5-lipo-oxygenase (ALOX5), TNF-α, colony-stimulating factor-2 (CSF2) and IL1-β, than adults ECFCs [85]. Moreover, CB-ECFCs display a reduced capacity to activate allogeneic mononuclear cells as reflected by reduced mRNA expression of *IL6*, *IL8*, *CSF2* and *ICOSL* compared to the one triggered by AB-ECFCs or mesenchymal cells. Surprisingly, CB-ECFCs exhibit a similar pattern of expression of HLA-ABC and HLA-DR antigens as mesenchymal stromal cells (MSCs), which are commonly transplanted irrespective of HLA-matching [85]. The concomitant expression of immunoregulatory molecules on CB-ECFCs such as HLA-G, a hallmark of cord blood-derived cells [86], may participate to their low immunogenicity. Indeed, this concept has been corroborated by the findings of Proust et al. who demonstrated that CB-ECFCs significantly inhibit PBMC allo-proliferation, unlike the HLA-II + lymphoblastoid cell line [87]. Mechanistically, CB-ECFCs highly expressed the immunosuppressive markers HLA-G, IL-10, and TGF-β1, allowing them to be tolerated and to promote a functional vascular network in immunocompetent mice. IL-10 has been shown to increase HLA-G mRNA levels in trophoblast cells [88] which promotes foeto-maternal tolerance by inhibiting maternal NK cells activation without impairing their antiviral immune function [89].

Altogether, these aforementioned immunomodulatory properties may explain the safety of ECFCs transplantation in several immunocompetent experimental animal models, without being rejected [90–92].

**Fig. 2:**
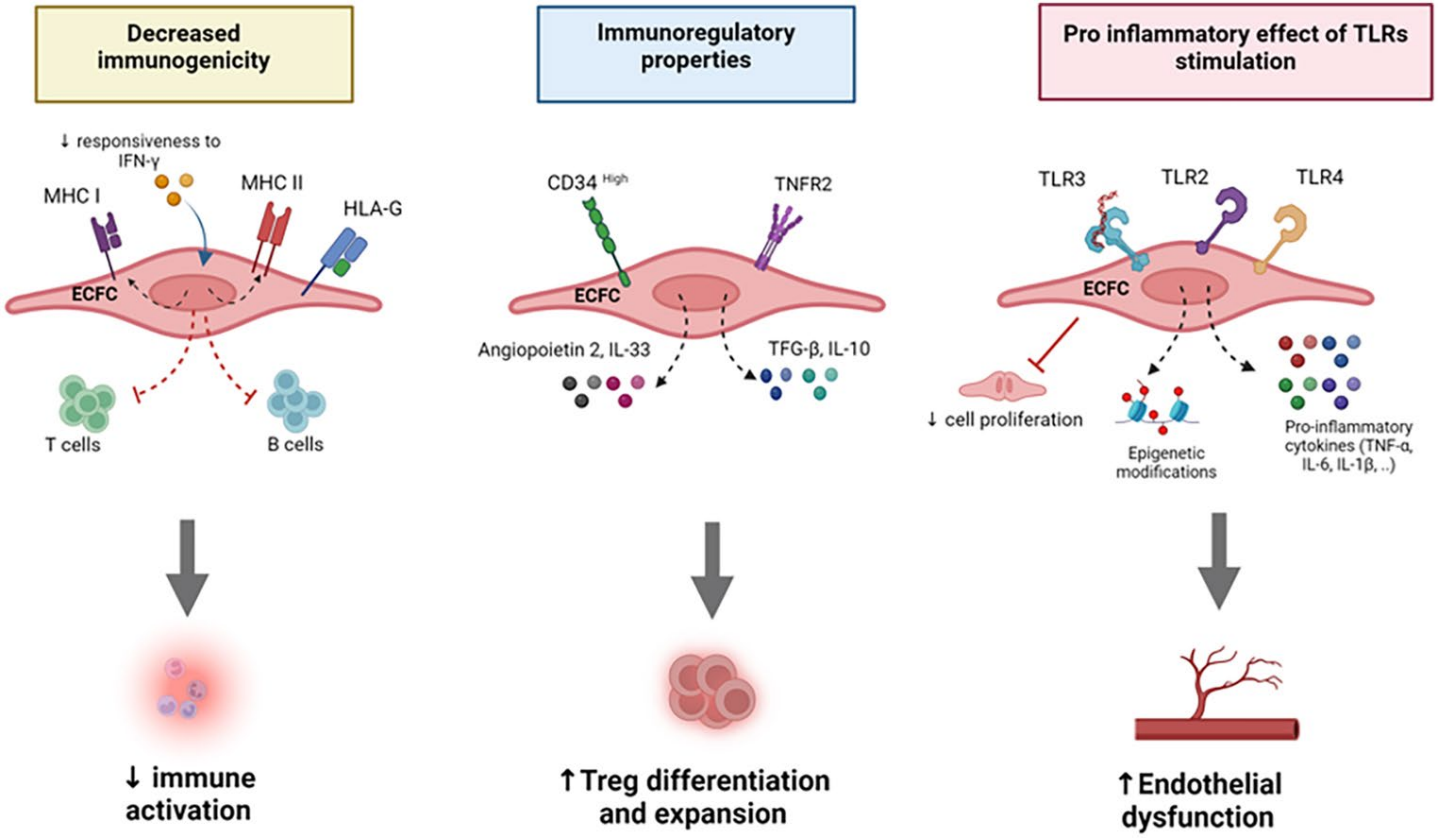
Immunomodulatory properties of ECFCs. ECFCs, especially those derived from cord blood, demonstrate low immunogenicity, characterized by reduced expression of MHC class I and II molecules, thereby minimizing immune recognition and rejection. This feature is enhanced by their capacity to express immunoregulatory molecules such as IL-10, IL-33, and HLA-G, contributing to their ability to promote immune tolerance and facilitate tissue repair. These cells express a variety of Toll-like receptors (TLRs), particularly TLR2, TLR3, and TLR4, which influence angiogenic processes according to the antigenic ligand present in the inflammatory microenvironment. Additionally, the expression of TNFR2 further supports their role in modulating immune response to inflammatory stimuli

In addition to their immunoregulatory function, a recent study demonstrated that ECFCs exhibit adaptive-like immune features and can establish inflammatory memory upon initial exposure to the viral-like ligand Poly(I: C) that stimulates TLR3 pathway [93]. A secondary stimulation of pre-exposed Poly(I: C) ECFCs induced robust changes in DNA methylation patterns at promoter genes related to inflammatory and immunometabolism pathways, a reminiscent observation of the trained immunity phenomenon [94, 95]. Moreover, the ECFCs harbored a distinct enrichment of the E74-like factor 1 (ELF1), a transcription factor that regulates the antiviral immune response and is distinct from the type I interferon signaling pathway [96]. However, further investigations are required to show the functional translation of the remodeled transcriptome and epigenome following Poly(I: C) pre-exposure of the ECFCs. A crosstalk has been described between the epigenetic landscape and the differential expression of microRNA (miRNA) cellular levels through the regulation of DNA methylation modifications [97]. Although Grelier et al. observed upregulation of miR-29b, miR-146a, and miR-155 expression following Poly(I: C) stimulation of ECFCs, selectively inhibiting these microRNAs did not alter the cells’ phenotype or function [72]. This reflects the complexity of ECFC biology at the transcriptomic and functional levels and highlights the need for future studies to decipher the molecular network involved in their homeostasis. Collectively, these data highlight the immunomodulatory functions of ECFCs (Fig. 2) and suggest that in addition to their vasculogenic capacity, these cells can closely interact will the immune microenvironment to elicit a tolerogenic state, a feature that can be affected by inflammatory stimuli.

## ECFCs: a promising approach for engineered organ revascularization (Fig. 3)

**Fig. 3:**
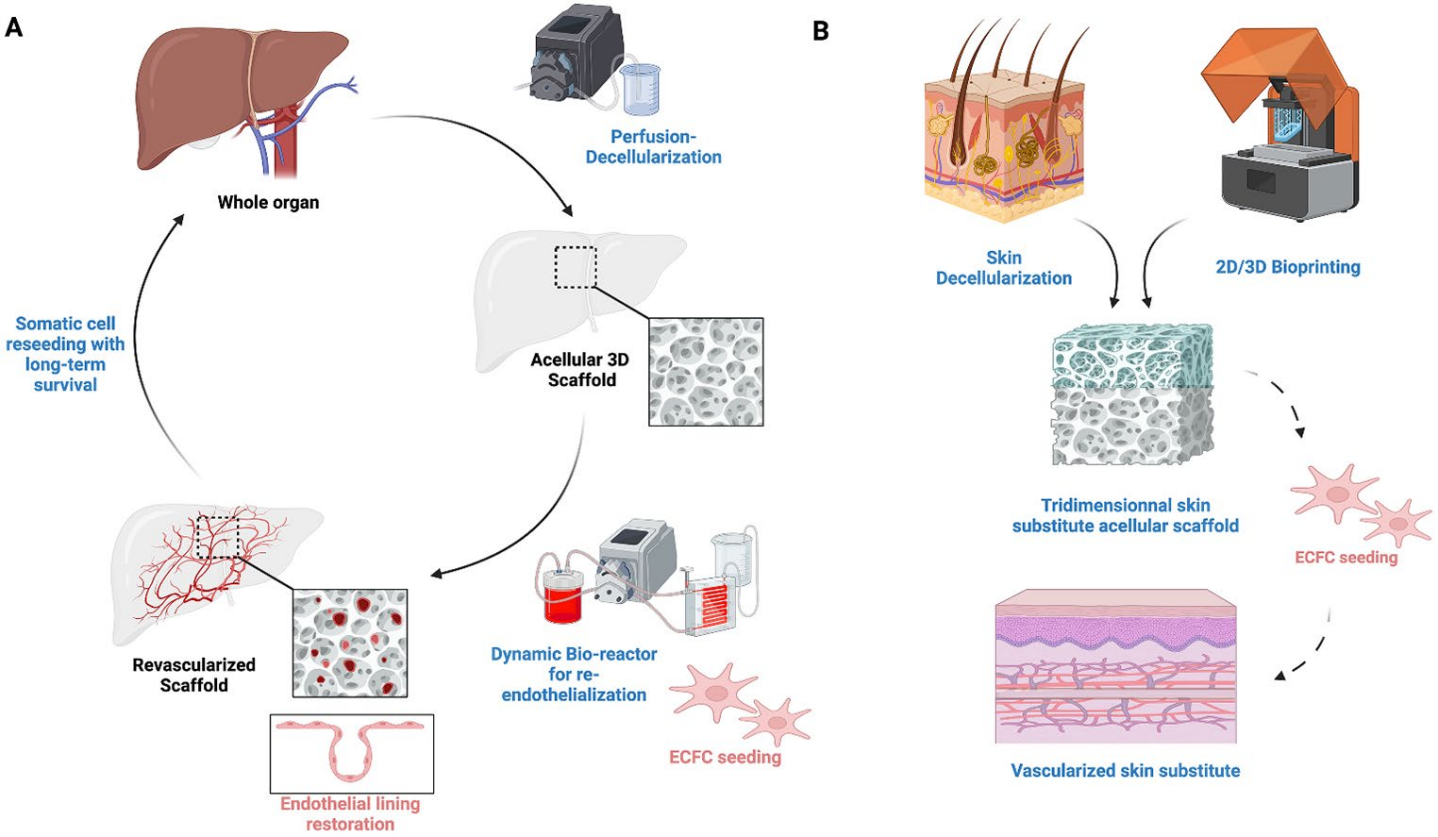
ECFCs are a promising approach for engineered organ revascularization. **A** Whole organ engineering consists of creating tridimensional acellular scaffolds for autologous or compatible cell seeing. The revascularization is the most critical step since it enables long-term survival of the seeded cells by providing blood flow through the whole scaffold. ECFCs hold promise for re-endothelialization of engineered organs through their multipotency, stemness and decreased immunogenicity. **B** Skin substitute prevascularization enhances integration within the host tissues. These engineered alternatives to skin grafts allow treating extended skin defects with low morbidity. Prevascularization demonstrated better integration within the recipient site. Tridimensional vascular networks could be obtained in vitro through ECFCs seeding

Tissue and organ engineering emerged in the 80’s as a promising alternative to allotransplantation. It aims to restore a function by building anatomical structures from acellular matrices and autologous or compatible cells. Several techniques exist to create tridimensional matrices (such as decellularization and 3D bioprinting) with encouraging results demonstrating the biocompatibility and absence of immunogenicity [98]. Growth factors such as Vascular Endothelial Growth Factor A (VEGF-A) and Epidermal Growth Factor (EGF) have been used to increase in vitro cell survival and multiplication within the scaffold, as well as mechanical stimulation such as shear forces and gravity. The progressive advances allowed great steps towards whole organ engineering, with promising results in liver, skin, lung and heart engineering. Moreover, the young field of Vascularized Composite Allotransplantation (VCA) has led to the development of reconstructive-aiming tissue engineering [99]. Building complex tissues would allow universal reconstructions in complex cases with no need for an immunosuppressive regimen, which is currently limiting VCA procedures. Multiple somatic cell seeding techniques have been described, leading to the successful colonization of tridimensional scaffolds [100]. However, to date, the main challenge for the prolonged success of these procedures is matrix revascularization. If simple contact and imbibition seem sufficient for thin acellular biostructures such as Acellular Dermal Matrices (ADMs), complex 3D scaffolds require parenchymal vascular tree restoration [101]. Similarly, novel engineered skin constructs are rapidly developing through multi-layer 3D bioprinting, increasing the need for adequate revascularization processes [102]. In decellularized scaffolds, authors have demonstrated the preservation of the vascular lumen, which could be used as an optimal bed for vessel regrowth. Still, no approach has demonstrated successful long-term results in sizeable scaffold re-endothelialization yet. Jank et al. demonstrated skin flap scaffold building with preservation of the vascular architecture, but pointed out the crucial importance of successful preparation of the vessels for re-endothelialization [103].

The evidence of using ECFCs for engineered construct revascularization remains poor to date. Cornerstone studies proved that ECFC seeding in collagen, hydrogels or matrigel implant [5, 29, 51, 62, 104, 105] were able to form functional blood vessels. Denecke et al. described in 2013 optimized ECFC culture on synthetic scaffold biomaterial, basing the culture media on human platelet lysate-enriched EGM-2 [106]. Their results showed minor endothelial tube formation and discrete adherence to the synthetic scaffold, but these encouraging results remain preliminary. It seems crucial to study evidence from alternative endothelial progenitors. Multiple attempts based on using Human Umbilical Vein Endothelial Cells (HUVECs) for acellular scaffold revascularization have been described. Hao et al. [107] experienced endothelial seeding using HUVECs in decellularized pancreatic scaffolds. While they showed a better potential for tube formation *versus* regular collagen scaffolds and demonstrated higher upregulation of pro-angiogenic factors such as MMP2, VEGF-A, and PAR-1 in endothelial cells cultured within decellularized scaffolds, they did not demonstrate 3D sprouting of vascular structures 24 h post-seeding. Watanabe et al. studied liver scaffold HUVEC-based revascularization and showed a partial coating of the portal vein sinusoids [108]. However, the endothelial cell phenotype was not studied despite being critical due to the sinusoid type found in the liver. Interestingly, they proposed using a perfusion machine to stimulate vessel tubulation through physiological-like shear forces. Despite this principle being highly promising, the lack of integrity of the endothelium lining led to major leakage that could affect the scaffold architecture [108]. Shaheen et al. produced pioneering results with successful perfusion-revascularization of full-sized liver scaffolds using HUVECs, with sinusoid-like characteristics [109]. Their technology was tested in vivo in an auxiliary allotransplantation model, showing endothelial immunogenicity. However, the perfusion and histological analysis of the reimplanted bioengineered livers showed a decreasing perfused and total graft volume, probably due to delayed thrombosis within the re-endothelialized vessels. The graft failure was significantly delayed by immunosuppressive drugs, emphasizing the importance of the immune reaction on the endothelial function. These results suggest the potential of HUVECs as a source for whole organ scaffold revascularization but highlight several limitations. Similarly, Yuan et al. used cultured Pulmonary Microvascular Endothelial Cells (PMECs) to achieve lung scaffold re-endothelialization [110]. They promoted cell migration and retention using a ROCK inhibitor to reduce cell size and facilitate intracapillary migration and used a machine-perfusion powered bioreactor to achieve intraluminal culture. Advanced TEM demonstrated lumen patency and endothelial coating, but the absence of type 1 alveolar epithelial cells and supporting vascular cells, such as pericytes and smooth muscle cells, prevented the full reconstruction of lung barrier functions. Although endothelial cells partially regained native phenotypes, their profiles remained incomplete, particularly for capillary subtypes. Their findings also missed key factors such as cyclic mechanical stretch, oxygen gradients, and paracrine signals, limiting biomimicry. Luo et al. achieved vascular construct re-endothelialization using a similar shear-stress enhanced bioreactor-based culture to enhance hiPSC-ECs alignment and adhesion [111]. Their engineered constructs demonstrated in vivo persistent patency and thrombosis prevention compared to non-endothelialized controls. These results were confirmed by Park et al. in a 2025 study using hiPSC-ECs and similar seeding techniques, with early evidence of smooth muscle recellularization and maturation [112].

The alternative of using stem/progenitor cells as a source of engineered organ revascularization potentially presents multiple advantages. Besides a higher potential of proliferation, which appears necessary due to the complex structure of a whole organ vascular tree, stem/progenitor cells can also lead to recreating the vascular microenvironment with supporting cell differentiation [113, 114]. Zhang et al. used a combination of human Adipose Stromal Cells (hASCs) and HUVECs for flap scaffold revascularization, which allowed successful microvascular implantation in nude rats [115]. They suggest the potential of hASCs towards an M2 macrophage phenotype, promoting an anti-inflammatory and immunotolerant environment. A recent study by Jiang et al. demonstrated successful 3D-printed scaffold revascularization using ESCs and ESC-derived vascular progenitor cells, with optimal integration to the recipient’s vasculature [116]. However, both their studies used nude rodent models and small constructs, limiting the interpretation to whole organ engineering with potential immunogenicity of ASC and ESC-derived vascular structures.

Using ECFCs in similar applications could significantly enhance vascular network formation within complex three-dimensional organ scaffolds suitable for clinical implantation. Indeed, their superior angiogenic potential, stable phenotype, and reduced immunogenicity would represent an asset. Specifically, CB-ECFCs exhibit greater angiogenic properties compared to HUVECs, endothelial cells derived from human embryonic stem cells or AB-ECFCs [5, 47–49, 52]. In artificial skin development, two main strategies prevail: the first involves artificially recreating skin using 2D/3D cell cultures, with or without 3D printing, and then implanting the bioengineered skin into the body to integrate with host tissue and promote vascularization. To attend to this objective, on the basis of previously skin organoids [117, 118], we developed a new biomaterial comprising a porous resorbable matrix [119] able to receive several cell subtypes. The porosity allows the material to have a structure similar to natural tissues, promoting cellular growth and integration when implanted into the body. This material will gradually dissolve or be absorbed by the surrounding skin over time, eliminating the need for a second surgery to remove it. The second strategy is decellularizing fresh skin for in vivo recellularization. In both approaches, enhancing the vascularization is crucial to ensure optimal engraftment. The use of ECFCs for simpler constructs, such as skin substitute pre-vascularization, has revealed successful blood vessel integration with enhanced healing [120]. If the challenge of skin substitute revascularization seems more affordable due to its decreased thickness and layered structure, this growing field is of crucial importance for addressing extended wounds as found in severe burns. ECFCs present a robust alternative for achieving enhanced outcomes through successful vascular tube formation and improved integration during the process of vascular self-sufficiency [121]. They demonstrate higher survival and proliferation potential [12, 47–49] and vasculogenic capability in vivo [51], along with immune privileges described here and stemness properties [39, 122, 123], making them an ideal cell type for pre vascularized organs or scaffolds.

## Conclusion

All in all, this review highlights the unique vasculogenic properties and therapeutic potential of CB-ECFCs in regenerative medicine. ECFCs from cord blood exhibit significant advantages over other sources, including higher proliferative capacity, robust vasculogenic potential, and fewer ethical concerns compared to embryonic stem cells. Additionally, these cells demonstrate immune-privileged characteristics, reducing the likelihood of immune rejection and improving the success rates of allogenic cell therapies. These properties result from a dampened proinflammatory profile, higher immunoregulatory capabilities, and an enhanced capacity to retain memory from previous antigenic exposures compared to mature or adult endothelial cells. Thus CB-ECFCs represent a promising avenue for cell-based therapies, offering a combination of vasculogenic potential, immune privilege, and therapeutic versatility. Transcriptomic, phenotypic and functional studies are warranted to provide a comprehensive analysis of the immunological features of CB-ECFCs. Moreover, future research and clinical applications should focus on harnessing these vasculogenic and immunomodulatory properties to develop effective treatments for cardiovascular conditions, but also on developing pre-vascularized organs.

## Data Availability

No datasets were generated or analysed during the current study.
